# TLR2 Promotes Monocyte/Macrophage Recruitment Into the Liver and Microabscess Formation to Limit the Spread of *Listeria Monocytogenes*

**DOI:** 10.3389/fimmu.2019.01388

**Published:** 2019-06-26

**Authors:** Guan Wang, Huajun Zhao, Bingqing Zheng, Dongxuan Li, Yi Yuan, Qiuju Han, Zhigang Tian, Jian Zhang

**Affiliations:** ^1^School of Pharmaceutical Sciences, Institute of Immunopharmaceutical Sciences, Shandong University, Jinan, China; ^2^School of Life Sciences, University of Science and Technology of China, Hefei, China

**Keywords:** TLR2, *Listeria monocytogenes*, macrophage, hepatocyte, migration

## Abstract

TLR2 signaling plays a critical protective role against acute *Listeria monocytogenes* (Lm) infection by up-regulating inflammatory cytokines and promoting macrophage antimicrobial capabilities. However, the underlying mechanism by which TLR2 regulates hepatic macrophage-mediated anti-Lm immune responses remains poorly understood. In this study, we found that both the absolute number and proportion of monocyte/macrophage (Mo/MΦ) in the liver and spleen of *Tlr2*^−/−^ mice were significantly lower compared to wild type mice. Changes in TLR2 signaling in both hepatocytes and Mo/MΦs were associated with the infiltration of Mo/MΦs in response to Lm-infection. Analyses by proteome profiler array and ELISA revealed that hepatocytes recruited Mo/MΦs via TLR2-dependent secretion of CCL2 and CXCL1, which was confirmed by receptor blocking and exogenous chemokine administration. Importantly, we found that TLR2 contributed to macrophage mobility in the liver through a TLR2/NO/F-actin pathway, facilitating the formation of macrophage-associated hepatic microabscesses. Moreover, TLR2 activation induced the expression of several PRRs on hepatic macrophages associated with the recognition of Lm and augmented macrophage bacterial clearance activity. Our findings provide insight into the intrinsic mechanisms of TLR2-induced Mo/MΦ migration and mobility, as well as the interaction between macrophages and hepatocytes in resistance to Lm infection.

## Introduction

*Listeria monocytogenes* (*L. monocytogenes*, Lm) is a foodborne Gram-positive intracellular pathogen capable of invasion and replication in both phagocytic and non-phagocytic cells (e.g., hepatocytes) (1–3). Overloaded intracellular Lm egresses and enters into the circulation, and finally invades the brain or the placenta, causing meningitis, encephalitis, septicemia, and even death (4, 5).

TLR3, TLR4, and TLR9 have been reported as being dispensable for Lm-induced immune activation (6). An early study showed that while MyD88 was necessary for resistance to Lm infection, a TLR2 deficiency did not influence the *in vivo* propagation of Lm (7). However, several subsequent studies have demonstrated that TLR2 is required for optimum control of Lm infection both *in vitro* and *in vivo* by enhancing the phagocytic ability of macrophages and up-regulating the production of TNF-α, IL-12, NO, as well as the expression of costimulatory molecules CD40 and CD86 (6, 8, 9).

Macrophages are vital immune cells associated with acute Lm infection. Kupffer cells (KCs), the liver resident macrophages, are the first cell type in the liver to be infected by Lm (10). KCs undergo rapid necroptotic cell death induced by the bacterial virulence factor listeriolysin O, and thus they cannot directly contribute to immunity or tissue repair (10). KC necrosis triggers the recruitment of circulating monocytes, which differentiate into monocyte-derived macrophages to compensate for KC loss (10, 11). Monocytes emigrate from circulation into Lm infected sites, which is triggered by CCL2 (MCP-1)- and CCL7 (MCP-3)-mediated stimulation of CCR2 on monocytes (12–15).

Given the importance of both TLR2 and macrophages for protecting against Lm infection, in the present study, we investigated the role of TLR2 in hepatic macrophage-mediated anti-Lm immune responses and explored how TLR2 affects monocyte/macrophage (Mo/MΦ) recruitment into the liver. We found that hepatocytes recruited Mo/MΦs by TLR2-induced CCL2 and CXCL1 secretion. In addition, TLR2/NO/F-actin pathway was involved in the regulation of macrophage mobilization in the liver, which contributed to the formation of microabscesses and limited the spread of Lm. These findings reveal the intrinsic mechanisms of TLR2 in resistance to Lm infection.

## Materials and Methods

### Bacterial Strain and Growth Condition

*L. monocytogenes* (ATCC 19114; a kind gift from Jinghua Yan, Institute of Microbiology Chinese Academy of Science, Beijing) was inoculated on Brain-Heart-Infusion (BHI) agar plates and cultured overnight at 37°C. After growing in BHI medium for 12 h, Lm was 1:100 diluted in fresh BHI medium and cultured to the mid-log growth phase for both *in vitro* and *in vivo* experiments.

### Mice and Lm Infection

Wild type (WT) C57BL/6 mice (HFK, Beijing), and *Tlr2*^−/−^ mice on a C57BL/6 background (kindly provided by Dr. Shaobo Su, Sun Yat-Sen University, Guangzhou, China) were housed in a pathogen-specific free facility. All animal protocols were approved by the Institutional Animal Care and Use Committee at Shandong University (LL-201602065) and met guidelines of the US National Institutes of Health for the humane care of animals. For the acute Lm infection model, 6 to 10-week-old male mice were i.p. injected with 1 × 10^6^ CFU Lm suspended in 200 μL of PBS.

### Cells Preparation

Hepatic mononuclear cells were separated by Percoll (GE Healthcare Life Sciences, Uppsala, Sweden) density gradient centrifugation as previously described (16). Mouse-derived primary hepatocytes were isolated and cultured using an established method (17). Briefly, mouse livers were perfused *in situ* with EGTA solution and digested with 0.075% collagenase type I (Gibco, CA, USA). Hepatocytes were washed and collected with low speed (50 × g) centrifugation, and then cultured in Dulbecco's modified Eagle medium (Gibco, NY, USA) containing 15% fetal bovine serum (FBS) for further experiments. Hepatocyte purity confirmed by microscopy and FACS was about 98% (Supplementary Figure S1A), and few (< 0.2%) of immune cells (CD45^+^) and endothelial cells (CD31^+^) were existed (Supplementary Figure S1B). For *in vitro* experiments involving Mo/MΦs, mice were i.p. injected with 2 mL 3% thioglycollate (Sigma-Aldrich, MO, USA) solution. Three days later, thioglycollate-elicited Mo/MΦs were obtained from the peritoneal fluids. After 1 h culture in RPMI 1640 medium (Gibco, NY, USA), cells that were not adhered were washed away. Over 95% of the thioglycollate-treated adherent peritoneal exudate cells (PECs) were Mo/MΦs. Then Mo/MΦs were cultured in RPMI 1640 medium containing 10% fetal bovine serum (FBS) for further *in vitro* studies.

### Bacterial Burden Measurement

Mice were infected with Lm for 3 days, and 100 μL blood was added to 1% Triton X-100 in PBS to release the intracellular bacteria. Tissues were removed from the infected mice and weighed before homogenization, and then lysed with 1% Triton X-100. Serial dilutions of lysates in PBS were plated on BHI agar plates. After 24 to 36 h of incubation at 37°C, the CFUs were counted.

To measure the macrophage bacterial burden, peritoneal macrophages were isolated and infected with Lm (MOI = 10:1) for 1 h *in vitro*, then treated with gentamicin (40 μg/mL) to kill any extracellular bacteria. For analysis of hepatocytes, hepatocytes were isolated from WT and *Tlr2*^−/−^ mice and infected with Lm (MOI = 20:1) for 4 h. The cells were harvested at the indicated time points and washed twice with PBS. CFUs were counted as described above.

### Flow Cytometry Analysis

Cells were incubated with anti-CD16/32 (clone: 93) (eBioscience, CA, USA) for 30 min and surface stained with a panel of antibodies at 4°C for 1 h. The following monoclonal antibodies were used in this study: anti-CD11b (M1/70), anti-F4/80 (BM8), and anti-CD169 (SER-4) were purchased from eBioscience, anti-CD45 (clone104) and anti-CD31 (clone390) were purchased from BioLegend (CA, USA), and anti-CD204 (REA148) was purchased from Miltenyi Biotec (Bergisch Gladbach, Germany). For intracellular CD206 staining, the cells were fixed and permeabilized following intracellular staining with anti-CD206 (C068C2, BioLegend, CA, USA) at 4°C for 1 h. For the staining of Ki-67, the cells were stained for surface markers, then fixed and permeabilized for intracellular anti-Ki-67 (16A8, BioLegend) staining using fixation and permeabilization buffers (eBioscience) according to the manufacturer's instructions. For ROS measurement, the cells were incubated with 10 μM Dichloro-dihydro-fluorescein diacetate (DCFH-DA) (Sigma-Aldrich, MO, USA) at 37°C for 20 min. All FACS data were acquired on a FACSCalibur (BD Biosciences) or AriaIII (BD Biosciences) flow cytometer and analyzed using FCS Express V3 and FlowJo 7.6 software.

### Macrophage Depletion and Transference

For macrophage depletion, WT or *Tlr2*^−/−^ mice were intravenously injected with 200 μL clodronate liposomes (Nico van RooijenLeb, Holland) 2 days before the cell transfer. Then, 2 × 10^6^ thioglycollate-elicited peritoneal exudate cells (PECs) were transferred to these mice via the tail vein. The following day, the mice were i.p. infected with 1 × 10^6^ CFU of Lm, and survival was monitored every day after infection.

### Cell Migration Assay

A transwell chamber migration assay was used to study cellular migration, chemotaxis, and mobility. For the recruiting ability analysis, hepatocytes were isolated from WT and *Tlr2*^−/−^ mice and seeded in a 24-well plate at density of 2.5 × 10^5^ cells/well. After 4 h of Lm infection (MOI = 20:1), the cells were cultured in 600 μL of fresh DMEM media with 15% FBS for 12 h, and gentamicin was added to kill any extracellular bacteria. In some experiments, hepatocytes were stimulated with 1 μg/mL Pam3CSK4 (InvivoGen, CA, USA) or PBS for 8 h, then the stimulation was removed and cultured in fresh DMEM for 12 h. Mo/MΦs with or without 50 nM CXCR2 antagonist SB225002 (Selleck, MA, USA) were plated in 100 μL of serum-free RPMI 1640 media with 2 × 10^5^ cells per transwell (8.0 μm, Corning, ME, USA). After 1 h incubation, cells in the inserts were allowed to transfer into the lower chamber for 4 h. At the end of the incubation, non-migrated cells on the upper surface were completely removed with a cotton swab. Cells that had migrated to the lower surface of the insert were fixed with methanol and stained with 0.1% crystal violet. Photographs were captured under a microscope (IX71, Olympus, Japan) and the migrated cells were counted in four random fields.

For the chemotaxis assay, 2 × 10^5^ Mo/MΦs were seeded in the inserts with 100 μL of serum-free RPMI 1640 media. The lower chamber contained 600 μL RPMI 1640 media with 10% FBS, and 10 ng/mL CCL2 (Peprotech) or 20 ng/mL CXCL1 (Peprotech) was added. After a 4 h incubation, the migrated cells were counted.

Cell mobility assays were also performed using transwell chambers. Mice were i.p. infected with 1 × 10^6^ CFU of Lm for 1 day, and peritoneal macrophages were added to the upper well at a density of 2 × 10^5^ cells/well. To confirm the effects of NO on the mobility of macrophages, 2 × 10^5^ peritoneal macrophages were stimulated with SNP (NO donor, Beyotime, China), or pre-treated with 100 μM NO scavenger Carboxy-PTIO (Beyotime, China) for 2 h in the insert and then stimulated with 100 ng/mL Pam3CSK4 (InvivoGen, CA, USA). These cells in the upper wells containing 100 μL serum-free media migrated to the lower chambers which contained 600 μL of RPMI 1640 media with 10% FBS for 4 h. Migrated cells were counted as described above.

To study vivo Mo/MΦ migration, WT and *Tlr2*^−/−^ mice were i.p. injected with 5 × 10^6^ CFSE-labeled (1 mM, Molecular Probes, Beyotime, Shanghai, China) thioglycollate-elicited PECs and 1 × 10^6^ CFU of Lm. Four hours later, the frequency and the absolute number of transferred Mo/MΦs (CFSE^+^ F4/80^+^) were detected.

### Histopathological Analyses

Livers were fixed in 4% paraformaldehyde (PFA) and embedded in paraffin. Tissue slices were sectioned to a thickness of 5 μm and stained with hematoxylin and eosin for histopathological evaluation.

### Immunohistochemistry and Immunofluorescence

Immunohistochemistry staining with anti-F4/80 or anti-Lm was performed as previously described (18). Briefly, the sections were blocked and incubated with anti-F4/80 (1:100 dilution; Santa Cruz, CA, USA) or anti-Lm (1:100 dilution; Abcam, Cambridge, UK) antibodies overnight at 4°C, washed, and then incubated with an HRP conjugated secondary antibody (1:200 dilution; ZSGB-BIO, China). Immunostaining was developed with diaminobenzidine (DAB) (ZSGB-BIO, China). The images were captured using a light microscope. The number of microabscesses was quantified by counting the lesions present in all fields on the section.

Intracellular bacteria present in Lm-infected hepatocytes were detected by Immunofluorescence. After *in vitro* Lm infection, the cells were fixed in 4% PFA and washed three times with PBS-T (PBS containing 0.1% Tween-20). The cells were blocked and incubated with an anti-Lm antibody (1:100 dilution) overnight at 4°C. After washing with PBS-T, cells were incubated with a DyLight 549-coupled secondary antibody (1:200 dilution; Abbkine, CA, USA) for 1 h. Diaminophenylindole (DAPI) (Beyotime, China) was used for nuclear staining. For hepatocytes identification by microscopy, anti-E-cadherin antibody (1:100 dilution; Santa Cruz, CA, USA) and Dapi were used.

Filamentous actin (F-actin) staining was performed as previously reported (19). Briefly, after each treatment, the cells were fixed with 4% PFA, then permeabilized and blocked with an immunofluorescence buffer (PBS containing 0.3% Triton X-100 and 1% BSA). The samples were stained with rhodamine-conjugated phalloidin (100 nM, Solarbio, China) for 1 h, then washed with PBS, and stained with DAPI for 5 min. The images were captured using a microscope (IX71, Olympus, Japan). The level of Lm and F-actin staining was analyzed by integrating the optical density (IOD) using Image-Pro Plus software.

### Cytokine/Chemokine Analysis and Nitric Oxide (NO) Quantification

WT and *Tlr2*^−/−^ mice were i.p. infected with 1 × 10^6^ CFU of Lm for 2 days. The level of cytokines/chemokines in the liver homogenates was measured by a proteome profiler array (ARY006, R&D Systems, MN, USA) according to the manufacturer's manual. Hepatocytes were isolated from WT and *Tlr2*^−/−^ mice and infected with Lm (MOI = 20:1) *in vitro*. After 12 h, the supernatant was harvested, and the level of chemokines was measured by an ELISA (eBioscience, CA, USA) according to the manufacturer's instructions. Peritoneal macrophages from WT mice were stimulated with different doses of Pam3CSK4 for 12 h. The level of NO in the supernatants was detected using a Nitric Oxide Synthase Assay Kit (Beyotime, China) according to the manufacturer's instructions.

### Apoptosis Analysis

To analyze apoptosis, hepatocytes were isolated from WT and *Tlr2*^−/−^ mice and seeded in 12-well plates. After 4 h of Lm infection (MOI = 20:1), the cells were incubated with fresh DMEM medium and gentamicin was added to kill any extracellular bacteria. The cells were harvested at the indicated time points and washed twice with PBS. Hepatocyte apoptosis was detected using an FITC Annexin V Apoptosis Detection Kit (BD Biosciences) according to the manufacturer's instructions.

### Real-Time Quantitative PCR

Total RNA was extracted from Mo/MΦs using TRIzol reagent (Invitrogen, Carlsband, CA, USA). cDNA was synthesized using 2.0 μg of the total RNA with M-MLV (Invitrogen) according to the manufacturer's instructions. The amplification of cDNA was performed by Real-time qPCR with SYBR Green Master (Roche, Indianapolis, IN, USA) on an iCycleriQ real-time PCR system (Bio-Rad, Hercules, CA, USA). The primers are listed in Supplementary Table S1. The PCR conditions were performed as described previously (20).

### Statistics

Statistical analyses were performed using GraphPad Prism Software. A standard two-tailed unpaired *t*-test or one-way ANOVA followed by a Bonferroni's *post-hoc* test was used for statistical analysis. For the survival curves, significance was determined with a log-rank (Mantel-Cox) test. The results are represented as the mean ± SEM. A value of *p* < 0.05 was considered statistically significant.

## Results

### TLR2 Deficiency Decreases Hepatic Mo/MΦ Infiltration in Response to Acute Lm Infection

**Figure 1:**
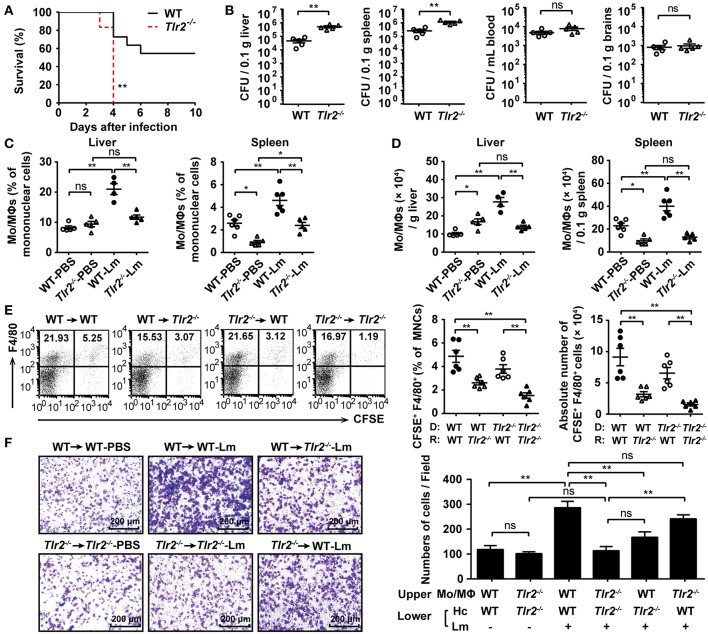
TLR2 deficiency decreases hepatic Mo/MΦ infiltration in response to acute Lm infection. **(A)** Age-matched WT and *Tlr2*^−/−^ mice were i.p. infected with 1 × 10^6^ CFU of Lm, and the survival rate of these mice was monitored (n ≥ 6, log-rank test, one representative of two independent experiments). **(B)** The bacterial burden was assessed by determining CFU numbers in the liver, spleen, blood, and brains 3 dpi (*n* = 5). **(C,D)** The frequency **(C)** and absolute number **(D)** of Mo/MΦs (CD11b^+^ F4/80^+^) in the liver and spleen 2 dpi (*n* = 5–6, one representative of two independent experiments). **(E)** WT and *Tlr2*^−/−^ mice were adoptively transferred with 5 × 10^6^ CFSE-labeled WT- or *Tlr2*^−/−^-PECs, separately, followed by Lm infection. The frequency and absolute number of transferred Mo/MΦs (CFSE^+^ F4/80^+^) were detected 4 h post-infection (*n* = 6). MNC, mononuclear cell; D, donor; R, recipient. **(F)** The ability of hepatocytes to recruit Mo/MΦs was measured using a transwell chamber migration assay (one representative of three independent experiments). Hc, hepatocyte. Data are presented as the mean ± SEM. ^*^p < 0.05, ^**^p < 0.01.

To verify the key role of TLR2 in the defense of an acute Lm infection, WT and *Tlr2*^−/−^ mice were i.p. infected with 1 × 10^6^ CFU of Lm. *Tlr2*^−/−^ mice exhibited a high sensitivity to Lm infection with high bacterial loads in both the liver and spleen, suggesting that TLR2 plays a critical role in the host immune response against Lm infection (Figures 1A,B). Mo/MΦs are the vital innate immune cells in response to an Lm infection. We analyzed the infiltration of Mo/MΦ in the liver and spleen after Lm infection (Supplementary Figure S2). As shown in Figures 1C,D, compared with WT uninfected mice, both the proportion and the absolute number of Mo/MΦs were increased in WT mice after infection, while a deficiency in TLR2 resulted in a slightly increase of Mo/MΦs and much less than that in WT-infected mice. It suggests that the activation of TLR2 affects the accumulation of Mo/MΦs in the liver and spleen.

The total number of liver macrophages increases upon Lm infection due to cellular proliferation and monocyte-derived macrophage compensation (11). The decreased number of Mo/MΦs in *Tlr2*^−/−^ mice was not the result of disturbed proliferative capacity, since the level of Ki-67 in Lm-infected *Tlr2*^−/−^-hepatic Mo/MΦs was similar to that in WT-hepatic Mo/MΦs (Supplementary Figure S3A). In addition, Kupffer cells undergone a rapid necroptosis upon 4 h of Lm infection, but there was no significant difference between WT and *Tlr2*^−/−^ mice (Supplementary Figure S3B).Therefore, we speculated that a TLR2 deficiency would impair the migratory ability of peripheral Mo/MΦs. To confirm this hypothesis, WT and *Tlr2*^−/−^ mice were transferred with CFSE-labeled WT- or *Tlr2*^−/−^ -PECs, respectively, and followed by Lm infection. Four hours later, we found that the frequency and absolute number of the transferred Mo/MΦs (CFSE^+^ F4/80^+^) in the liver of WT mice that received CFSE-labeled WT PECs was higher than that of the *Tlr2*^−/−^ mice that received CFSE-labeled *Tlr2*^−/−^ PECs (Figure 1E). Importantly, for the same donor, the WT recipient liver recruited more Mo/MΦs than that of the *Tlr2*^−/−^ recipient liver; for the same recipients, the frequency or absolute number of CFSE-labeled *Tlr2*^−/−^ -PECs showed a decrease tendency compared to CFSE-labeled WT- PECs, but there was no significant difference, indicating that TLR2 expressed on liver tissue contributed to the recruitment of Mo/MΦs to some extent. Hepatocytes represent a major component of liver tissue, which can be infected by Lm (Supplementary Figure S3C). We found the bacterial burden and rate of hepatocyte apoptosis were similar between Lm-infected WT- and *Tlr2*^−/−^-hepatocytes (Supplementary Figures S3D,E). However, in the transwell chamber migration assay, we found that WT hepatocytes could effectively recruit Mo/MΦs during an Lm infection (WT → WT-Lm group), while the recruiting ability of *Tlr2*^−/−^ hepatocytes was impaired (*Tlr2*^−/−^→*Tlr2*^−/−^-Lm group). Similarly, WT-hepatocytes recruited more Mo/MΦs than *Tlr2*^−/−^-hepatocytes did (WT → WT-Lm vs. WT → *Tlr2*^−/−^-Lm, *Tlr2*^−/−^ → WT-Lm vs. *Tlr2*^−/−^→*Tlr2*^−/−^-Lm) (Figure 1F). In addition, the role of TLR2 on hepatocytes recruiting Mo/MΦs was confirmed by Pam3CSK4 stimulation. As shown in Supplementary Figure S3F, WT-hepatocytes pre-stimulated with Pam3CSK4 recruited more WT- Mo/MΦs than *Tlr2*^−/−^-hepatocytes. These findings indicate that TLR2 signaling in hepatocytes is associated with the infiltration of Mo/MΦs in response to Lm infection.

### TLR2 Promotes the Production of Chemokine Released by Hepatocytes in Response to Lm-Infection

**Figure 2:**
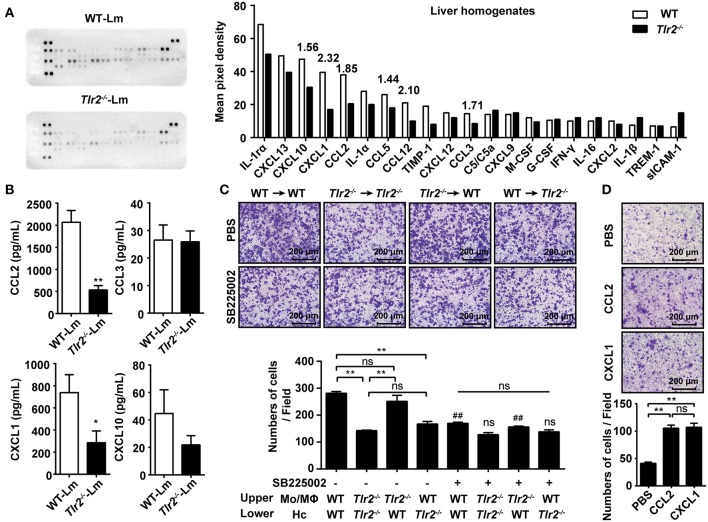
TLR2 promotes the production of chemokine released by hepatocytes in response to Lm-infection. **(A)** WT and *Tlr2*^−/−^ mice were i.p. infected with 1 × 10^6^ CFU of Lm for 2 days. The expression of cytokines/chemokines in the liver homogenates (pooled samples from five mice per group) was measured using a proteome profiler array. **(B)** Hepatocytes were isolated from WT and *Tlr2*^−/−^ mice and infected with Lm (MOI = 20:1) *in vitro*. Chemokine production was measured by an ELISA at 12 h post infection (*n* ≥ 5). **(C)** Hepatocytes from WT or *Tlr2*^−/−^ mice were seeded into the lower chamber and infected with Lm for 12 h. The Mo/MΦs were added to the inserts with or without the CXCR2 antagonist, SB225002. Photographs were taken after 4 h of migration (one representative of three independent experiments). #, SB225002-treated groups vs. untreated groups. **(D)** Mo/MΦ recruitment by CCL2 and CXCL1 was measured using a transwell chamber migration assay (one representative of three independent experiments). Data are presented as the mean ± SEM. ^*^p < 0.05, ^**^, ##*p* < 0.01.

Chemokines play a crucial role in Mo/MΦ recruitment mediated by hepatocytes. CCL2, CCL7, and CCL12 trigger Mo/MΦ migration from the circulation into Lm-infected sites via CCR2. In addition, CCL3, CCL4, and CCL5 are also involved in Mo/MΦ migration via CCR1 and CCR5 (21). Using a proteome profiler array, we analyzed the cytokine/chemokine profile of Lm-infected liver homogenates. The results revealed that the expression of CCL2, CCL3, CCL5, and CCL12 were lower in the *Tlr2*^−/−^ mice compared with WT mice, while CCL4 and CCL7 were undetectable (Figure 2A). Moreover, we found that the expression of chemokines associated with neutrophils and T cells, including CXCL1 and CXCL10, was also decreased in *Tlr2*^−/−^ mice. Further analysis revealed that the levels of *Ccl2, Ccl3, Cxcl1*, and *Cxcl10* in *Tlr2*^−/−^-hepatocytes 12 h following Lm-infection *in vitro* were substantially lower than that in WT-hepatocytes (Supplementary Figure S4A). Additionally, an ELISA analysis confirmed that the production of CCL2 and CXCL1 was reduced in the infected *Tlr2*^−/−^-hepatocytes compared to the infected WT-hepatocytes, while there was no statistical difference in CCL3 or CXCL10 production (Figure 2B). The levels of these chemokines did not show significant change in uninfected *Tlr2*^−/−^ hepatocytes compared to WT hepatocytes (Supplementary Figure S4B). Although IL-4 and IL-33 lead to Mo/MΦ recruitment (11), there was no significant difference between Lm-infected WT and *Tlr2*^−/−^ mice (Supplementary Figure S4C).

CCL2 is widely known to recruit Mo/MΦs; however, the role of CXCL1 in Mo/MΦ recruitment remains unknown. To confirm the role of CXCL1 in Mo/MΦ recruitment, the infiltration capacity of Mo/MΦs exposed to the CXCL1 receptor CXCR2 antagonist, SB225002, was analyzed using a transwell assay. Although the ability of WT-hepatocyte-mediated Mo/MΦ infiltration was obviously inhibited by SB225002, it had no significant influence on *Tlr2*^−/−^-hepatocytes (Figure 2C), indicating that CXCL1 contributed to TLR2-induced Mo/MΦ infiltration. In addition, the expression of *Cxcr2* was decreased on *Tlr2*^−/−^-Mo/MΦs (Supplementary Figure S4D), indicating that CXCL1-CXCR2 signaling was involved in TLR2-induced Mo/MΦ migration. Interestingly, a comparative analysis revealed that the chemotaxis ability was similar for both CCL2 and CXCL1 (Figure 2D), suggesting CXCL1 was as important as CCL2 for Mo/MΦ recruitment. These findings demonstrate that the activation of TLR2 signaling in response to Lm-infection promotes the production of CCL2 and CXCL1 by hepatocytes, recruiting Mo/MΦs into the liver.

### TLR2 Promotes Macrophage Mobility

**Figure 3:**
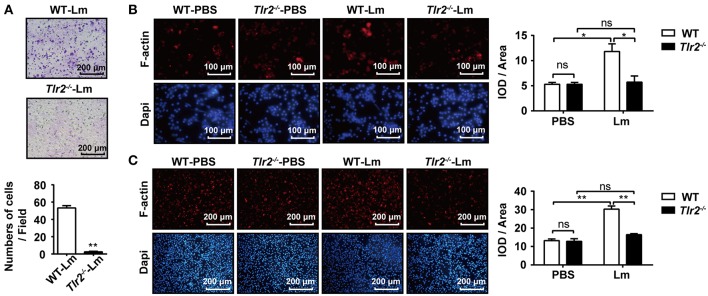
TLR2 promotes macrophage mobility. **(A)** WT and *Tlr2*^−/−^ mice were i.p. infected with 1 × 10^6^ CFU of Lm for 1 day. The mobility of peritoneal macrophages was measured using a transwell chamber migration assay. **(B,C)** Macrophages were isolated from 1 day infected or uninfected WT and *Tlr2*^−/−^ mice, and then seeded in 12-well plates at a density of 2 × 10^5^ cells/well. After 1 h, cells were fixed, permeabilized, and stained with rhodamine-conjugated phalloidin and DAPI **(B)**. WT and *Tlr2*^−/−^ macrophages were infected with Lm *in vitro* for 1 h. F-actin was stained with rhodamine-phalloidin **(C)**. The ratio of integrated optical density (IOD) to area of interest was calculated to assess the level of F-actin. Red, F-actin; blue, Dapi. One representative of three independent experiments. Data are presented as the mean ± SEM of three independent experiments. ^*^p < 0.05, ^**^p < 0.01.

Subsequently, we tested how TLR2 signaling promoted the mobility of macrophages. Using a transwell chamber migration assay, obvious migration was observed in Lm-infected WT macrophages (Figure 3A); however, few *Tlr2*^−/−^ macrophages migrated into the lower chamber, indicating that TLR2 triggered the mobility of macrophages. Since the polymerization of F-actin is the foundation of cellular motility, the level of F-actin in the macrophages derived from Lm-infected *Tlr2*^−/−^ mice was lower than in WT-macrophages (Figure 3B). Similar results were obtained from WT- and *Tlr2*^−/−^-macrophages infected with Lm *in vitro* (Figure 3C). These findings indicate that the activation of TLR2 promotes macrophage mobility in the liver by inducing the polymerization of F-actin.

### TLR2-NO Pathway Regulates F-Actin Polymerization

**Figure 4:**
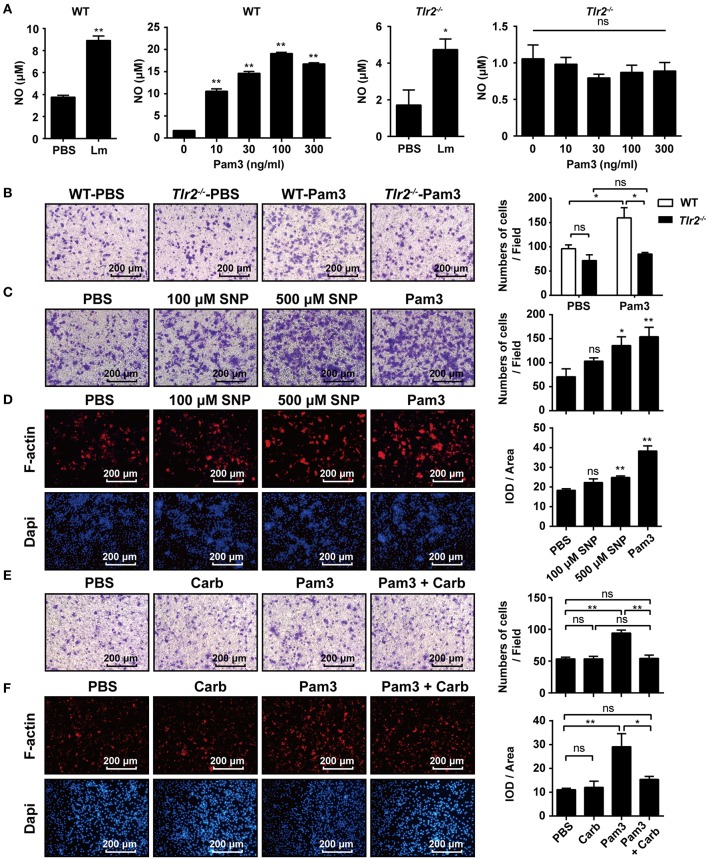
TLR2-NO pathway regulates F-actin polymerization. **(A)** Macrophages derived from WT and *Tlr2*^−/−^ mice were infected with Lm (MOI = 10:1) for 1 h *in vitro*, then treated with gentamicin and cultured for 12 h, or stimulated with different doses of Pam3CSK4 for 12 h. The level of NO in the supernatant was detected. **(B)** Macrophages from WT and *Tlr2*^−/−^ mice were stimulated with 100 ng/mL Pam3CSK4. The mobility of peritoneal macrophages was measured using a transwell chamber migration assay. **(C)** WT macrophages were added to the inserts and stimulated with SNP (NO donor), Pam3CSK4, or PBS as a control. Photographs were taken after 4 h of migration. **(D)** WT macrophages were stimulated with SNP, Pam3CSK4, or PBS as a control for 1 h. F-actin was detected by rhodamine-phalloidin staining. Red, F-actin; blue, Dapi. **(E,F)** WT macrophages were pre-treated with the NO scavenger, Carboxy-PTIO, or PBS as a control, then stimulated with Pam3CSK4. Cellular mobility was measured using a transwell chamber migration assay **(E)**, and F-actin was detected by rhodamine-phalloidin staining **(F)**. The ratio of integrated optical density (IOD) to area of interest was calculated to assess the level of F-actin **(D,F)**. Red, F-actin; blue, Dapi. B-F, one representative image of three independent experiments. Data are presented as the mean ± SEM of three independent experiments. ^*^p < 0.05, ^**^p < 0.01.

NO has been reported to promote the migration of cancer cells, endothelial cells, and keratinocytes (19, 22, 23). Similar to Lm, we found that the TLR2 agonist, Pam3CSK4, enhanced NO production in the supernatant of WT-macrophages in a dose-dependent manner, while *Tlr2*^−/−^-macrophages produced less NO under Lm infection than WT-macrophages and had no response to Pam3CSK4 stimulation (Figure 4A). Using a transwell chamber migration assay, we observed that WT- macrophages exhibited high mobility under Pam3CSK4 stimulation, whereas *Tlr2*^−/−^-macrophages did not elicit a significant response to Pam3CSK4 stimulation (Figure 4B). Furthermore, SNP (NO donor) also stimulated macrophage mobility (Figure 4C) and F-actin polymerization (Figure 4D). But, the macrophage mobility and F-actin polymerization in the macrophages induced by TLR2 could be suppressed by the NO scavenger, Carboxy-PTIO (Figures 4E,F). Compared to Pam3CSK4, SNP (NO donor) promoted the migration and F-actin polymerization of *Tlr2*^−/−^-macrophages (Supplementary Figure S5). These data indicate that TLR2 triggers macrophage mobility via the TLR2/NO/F-actin pathway.

### TLR2 Contributes to Hepatic Microabscess Formation

**Figure 5:**
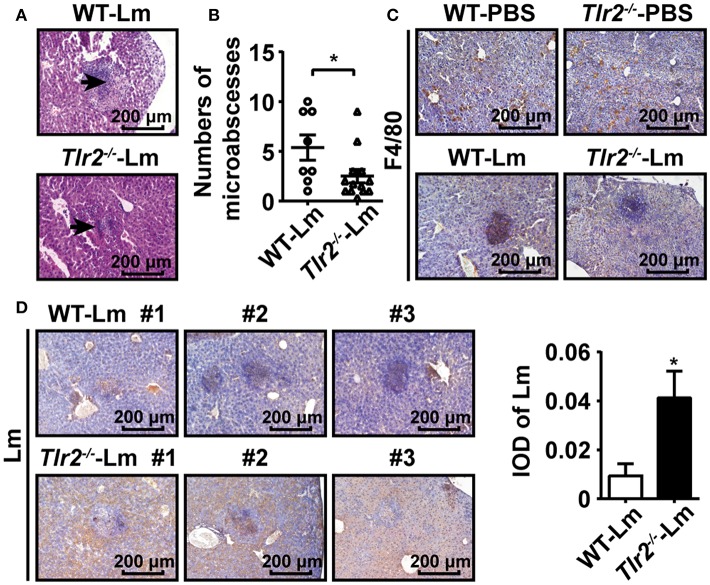
TLR2 contributes to hepatic microabscess formation. **(A)** Pathological sections (H&E staining) of livers obtained from Lm-infected WT and *Tlr2*^−/−^ mice 2 dpi. Black arrows indicate microabscesses. One representative image of three independent experiments is shown. **(B)** The total number of microabscesses in the liver of Lm-infected WT and *Tlr2*^−/−^ mice 2 dpi (*n* ≥ 8). The number of microabscesses was quantified by counting the lesions presented in 18 fields on each section. **(C,D)** IHC was performed for F4/80 **(C)** and Lm (**D**, left) in the livers of WT and *Tlr2*^−/−^ mice (*n* = 5). The level of Lm was analyzed by IOD (**D**, right). One representative image of three independent experiments. Data are presented as the mean ± SEM. ^*^p < 0.05.

During intracellular bacterial infection, immune cells (e.g., macrophages and neutrophils) are recruited into the site of infection by chemokines, which results in the formation of microabscesses to limit the spread of bacteria by phagocytosis (8). Therefore, we investigated the formation of hepatic microabscesses by analyzing the pathological sections 2 dpi. Our results showed that only a few hepatic microabscesses formed in *Tlr2*^−/−^ infected mice compared to WT infected mice (Figures 5A,B). To clarify whether TLR2 was involved in the formation of hepatic microabscesses via interfering with macrophage behavior, the distribution of macrophages in the livers of Lm-infected or uninfected WT and *Tlr2*^−/−^ mice was analyzed by immunohistochemistry (IHC). As shown in Figure 5C, both WT- and *Tlr2*^−/−^-macrophages were scattered evenly throughout the uninfected liver tissues. In response to Lm infection, WT-macrophages aggregated to form hepatic microabscesses, whereas most *Tlr2*^−/−^-macrophages remained in a distributed manner with few microabscesses. Importantly, IHC analysis revealed that Lm was limited in the hepatic microabscesses of WT mice, while Lm diffused and replicated in the livers of the *Tlr2*^−/−^ mice (Figure 5D). These results indicate that TLR2 enhances the formation of macrophage-associated hepatic microabscesses to limit Lm spread.

### TLR2 Plays a Critical Role in Macrophage-Mediated anti-lm Responses

**Figure 6:**
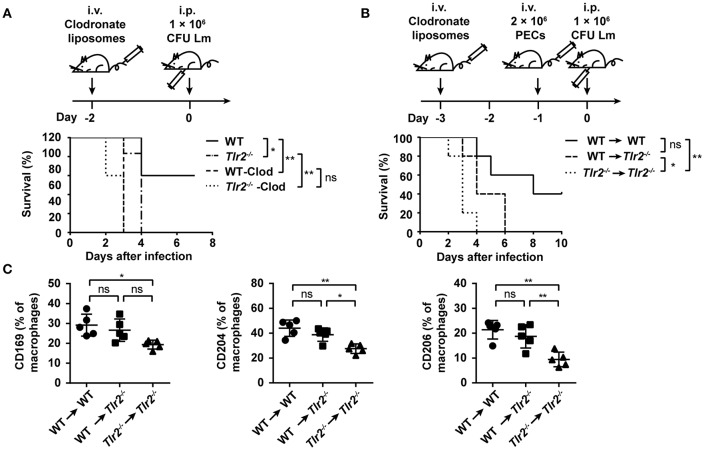
TLR2 plays a critical role in macrophage-mediated anti-Lm responses. **(A)** WT and *Tlr2*^−/−^ mice were treated with clodronate liposomes 2 days before Lm infection. Survival was monitored every day (*n* ≥ 5, log-rank test). **(B,C)** WT or *Tlr2*^−/−^ mice were treated with clodronate liposomes to deplete macrophages. Two days later, these mice were transferred with 2 × 10^6^ PECs from WT or *Tlr2*^−/−^ mice and subsequently infected with Lm the following day. The survival was monitored every day **(B)** (*n* = 5, log-rank test). The expression of CD169, CD204, and CD206 on macrophages (CD11b^+^ F4/80^+^) in the liver was detected by FACS 2 dpi **(C)** (*n* = 5). Data are presented as the mean ± SEM. ^*^p < 0.05, ^**^p < 0.01.

Finally, we sought to identify the crucial role of TLR2 on macrophage-mediated anti-Lm responses. Since WT and *Tlr2*^−/−^ mice were treated with clodronate liposomes to deplete macrophages (Supplementary Figure S6), followed by an Lm infection, these mice exhibited a similar sensitivity to Lm infection (Figure 6A). Furthermore, as shown in Figure 6B, the survival of *Tlr2*^−/−^ mice was significantly (*p* = 0.0437) prolonged by an adoptive transfer with WT-Mo/MΦs. These results suggest the importance of TLR2 in macrophage-mediated anti-Lm immune responses.

In an acute Lm infection, macrophages limit Lm spread by phagocytosis and bacterial clearance. We observed that a TLR2 deficiency decreased the recognition and phagocytosis of Lm by macrophages (Supplementary Figure S7A), which was accompanied by the low level of pattern recognition receptors (PRRs) associated with phagocytosis (e.g., CD169, CD204, and CD206) (Supplementary Figure S7B). However, the expression of CD169, CD204, and CD206 on macrophages transferred in *Tlr2*^−/−^ mice (WT → *Tlr2*^−/−^ group) was similar to that in WT mice (WT → WT group), indicating that TLR2 regulates the expression of these PRRs on macrophages (Figure 6C). Moreover, the expression of inducible nitric oxide synthase (iNOS) in *Tlr2*^−/−^-macrophages was far below that of the WT-macrophages on 1 dpi (Supplementary Figure S7C); however, there was no statistical difference in the expression of reactive oxygen species (ROS) (Supplementary Figure S7D). IFN-γ leads to Mo/MΦ activation, and IL-6 is produced by active Mo/MΦs. As shown in Supplementary Figure S7E, the level of IFN-γ and IL-6 in the serum of Lm-infected *Tlr2*^−/−^ mice was lower than in WT mice. Furthermore, following an *in vitro* infection with Lm for 12 h, the bacterial burden in *Tlr2*^−/−^-macrophages was higher than that in WT-macrophages (Supplementary Figure S7F). Therefore, these data indicate that TLR2 is associated with the phagocytosis and anti-Lm effects of macrophages.

## Discussion

The importance of TLR2 signaling in host resistance to Lm infection has been reported, with a focus on the up-regulation of inflammatory cytokines and costimulatory molecules, enhanced phagocytosis, and the promotion of the antimicrobial ability of macrophages (6, 8, 9). Our study further confirms the crucial role of TLR2 in host defense against Lm infection, as Lm-infected *Tlr2*^−/−^ mice were associated with poor survival and high bacterial loads in both the liver and spleen. Importantly, we found that *Tlr2*^−/−^ mice failed to recruit Mo/MΦs into the liver and spleen. Since Mo/MΦs play a critical role in the anti-Lm immune response, and the migration of Mo/MΦs from the circulation into Lm-infected sites is important for a Mo/MΦ-dependent immune response, we considered that an insufficient number of Mo/MΦs might be the primary reason for the poor survival of *Tlr2*^−/−^ mice.

The infiltration of peripheral Mo/MΦs is the main source of the increased hepatic Mo/MΦs in response to Lm infection. Moreover, CCL2 is considered to be the prototypical monocyte-attracting CC chemokine, which can be induced by a variety of stimuli in a TLR2/4/MyD88-dependent manner (13, 24). In the liver, hepatocytes, hepatic stellate cells, and KCs are the sources of CCL2 (25–27). In the present study, we confirmed that TLR2 can induce the production of CCL2 by hepatocytes. Another prominent chemokine pathway of monocytes is the CXCL10-CXCR3 interaction (28). Although we observed that the expression of CXCL10 was lower in *Tlr2*^−/−^ liver homogenates compared to that of WT mice, there was no statistical difference in the supernatants of Lm-infected *Tlr2*^−/−^ and WT hepatocytes. CXCL1 is a classical chemokine associated with neutrophil recruitment, and its receptor is CXCR2 (26, 29). Interestingly, we observed that the production of CXCL1 was decreased by a TLR2 deficiency. Further investigation revealed that the role of CXCL1 in Mo/MΦ recruitment was inhibited by a CXCR2 antagonist but enhanced by exogenous CXCL1 administration. In addition, the expression of *Cxcr2* was decreased on *Tlr2*^−/−^-Mo/MΦs. Therefore, TLR2 signaling regulated the expression of CXCL1 and CXCR2 on hepatocytes and Mo/MΦs, respectively. Thus, CXCL1-CXCR2 signaling provides a novel and important mechanism for TLR2-induced Mo/MΦ migration in addition to CCL2.

Macrophage mobility also contributed to the infiltration efficiency. We found that the deficiency of TLR2 on macrophages decreased the migration of macrophages by inhibiting cellular mobility. The polymerization of F-actin is a common element for cellular motility. Rho, Rac, and cdc42 mediate changes in the actin cytoskeleton, thereby affecting cellular motility (30). Here, we found that TLR2 can induce the polymerization of F-actin, enhancing macrophage mobility. Several studies have shown the regulatory effects of TLRs on the actin cytoskeleton (31, 32). For instance, Kleveta et al. demonstrated that paxillin and N-WASP are involved in the reorganization of the actin cytoskeleton in LPS-induced macrophage motility (31). Moreover, McGarry et al. suggested that TLR2-induced migration and invasion of primary rheumatoid arthritis synovial fibroblasts is partially mediated by the TLR2-β1-integrin-Rac1 pathway in cytoskeletal dynamics (32). However, the mechanism by which TLR signaling regulates the polymerization of F-actin remains unclear, as cytoskeletal remodeling is typically not the part of TLR pathway models (33).

The small GTPases, Cdc42, and Rac1 are effectors in the NO-HIF axis involved in RAW264.7 cell cytoskeleton reorganization and migration (34), and NO drives human keratinocyte cell migration by modulating the actin cytoskeleton through the cGMP-PKG-Rho GTPase signaling pathway (19). Given that TLR2 promotes the production of NO and the role of NO on the migration of cancer cells, we suspected that NO could be the connection between TLR2 and F-actin assembly. Indeed, under the stimulation of NO donor or TLR2 agonist, cellular mobility and F-actin polymerization were augmented, however the TLR2 induced F-actin polymerization could be dampened by the NO scavenger, Carboxy-PTIO, suggesting that TLR2 activation promotes macrophage mobility through the TLR2/NO/F-actin pathway.

Hepatic microabscesses are observed during the early stage of Lm infection, and consist of neutrophils, macrophages, as well as some T and B cells (8, 35, 36), which are regarded as a characteristic phenomenon associated with bacterial infection. Mice which were depleted macrophages (37) or specifically impaired NF-κB activation in hepatocytes (38) reported a loss of liver microabscess formation and an increased susceptibility to Lm infection. During Lm infection, we found that the scattered macrophages assembled into microabscesses in the presence of TLR2, and Lm in WT mice was limited to the microabscesses region. However, Lm diffused and replicated in the infected tissues of *Tlr2*^−/−^ mice, which would increase the sensitivity to Lm. In addition, Lm infection would lead to tissue injury by inducing leukocyte apoptosis (38–40). Since both tissue-resident and recruited macrophages can ingest apoptotic leukocytes (40, 41), therefore the insufficient number of Mo/MΦs in *Tlr2*^−/−^ mice would result in the deficiency of apoptotic leukocyte clearance and subsequent serious tissue injury, which would be another important reason for the sensitivity exhibited by *Tlr2*^−/−^ mice in response to Lm infection.

Macrophages limit bacterial spread by phagocytosis, which is crucial for the defense against Lm infection. Shen et al indicated that TLR2-MyD88-dependent signaling enhanced macrophage-mediated phagocytosis of Lm through the activation of PI3K and Rac1 (9). Here, we found that TLR2 activation induced the expression of several PRRs on hepatic macrophages, which might contribute to phagocytosis. For instance, CD169 (Siglec-1) contributes to viral recognition by sialic acids (42, 43), CD204 (Macrophage scavenger receptor 1) has been reported to be an important receptor involved in the phagocytosis of *Staphylococcus aureus* (44), and CD206 (macrophage mannose receptor 1) is well-known in the phagocytic and pinocytic uptake of sugar containing molecules (45). The up-regulation of these PRRs on hepatic macrophages by TLR2 activation likely also enhanced the phagocytosis of other pathogens during Lm infection. In addition, TLR2 contributed to the differentiation of monocytes and monocyte-derived macrophages into Kupffer cells (data not show), and promoted IL-6 and IFN-γ secretion, which would further help bacterial clearance.

Overall, as illustrated in Supplementary Figure S8, Lm-infected hepatocytes secrete chemokines (e.g., CCL2 and CXCL1) in a TLR2 dependent manner. CCL2 and CXCL1 function to recruit monocytes/macrophages into the liver. Then, the activation of TLR2 on macrophages promotes cellular mobility through the TLR2/NO/F-actin pathway. Finally, macrophages participate in the formation of hepatic microabscesses and limit Lm spread by TLR2-induced phagocytosis and bacterial clearance. Our study clarified the intrinsic mechanisms of TLR2 involved in resistance to Lm infection, especially in monocyte/macrophage recruitment into the liver and hepatic microabscess formation.

## Ethics Statement

This study was carried out in accordance with the recommendations of the guidelines of humane care of animals of Shandong University, and met the guidelines of the US National Institutes of Health for the humane care of animals. The protocol was approved by the Institutional Animal Care and Use Committee at Shandong University.

## Author Contributions

JZ, GW, QH, and ZT conceived and designed the experiments. GW, HZ, BZ, DL, and YY performed the experiments. GW and JZ analyzed the data. GW and JZ wrote the paper. All authors critically evaluated the written manuscript and had final approval of the manuscript.

### Conflict of Interest Statement

The authors declare that the research was conducted in the absence of any commercial or financial relationships that could be construed as a potential conflict of interest.
