# Supplementary material for: TLR2 Promotes Monocyte/Macrophage Recruitment Into the Liver and Microabscess Formation to Limit the Spread of Listeria Monocytogenes
Source: Front Immunol. 2019 Jun 26;10:1388. doi: 10.3389/fimmu.2019.01388 (PMC6607897; doi:10.3389/fimmu.2019.01388)
Supplement: Supplementary file 1 [file Data_Sheet_1.pdf]

Supplementary Material

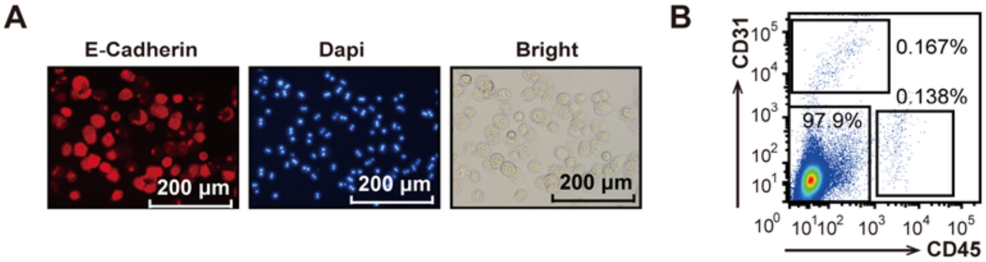

Supplementary Figure S1. Hepatocyte isolation and identification. (A) Hepatocytes were isolated from WT mice and stained with E-Cadherin and Dapi. (B) The hepatocyte purity was detected by FACS (one representative image of three independent experiments). $CD45^+$ cells, immune cells; $CD31^+$ cells, endothelial cells.

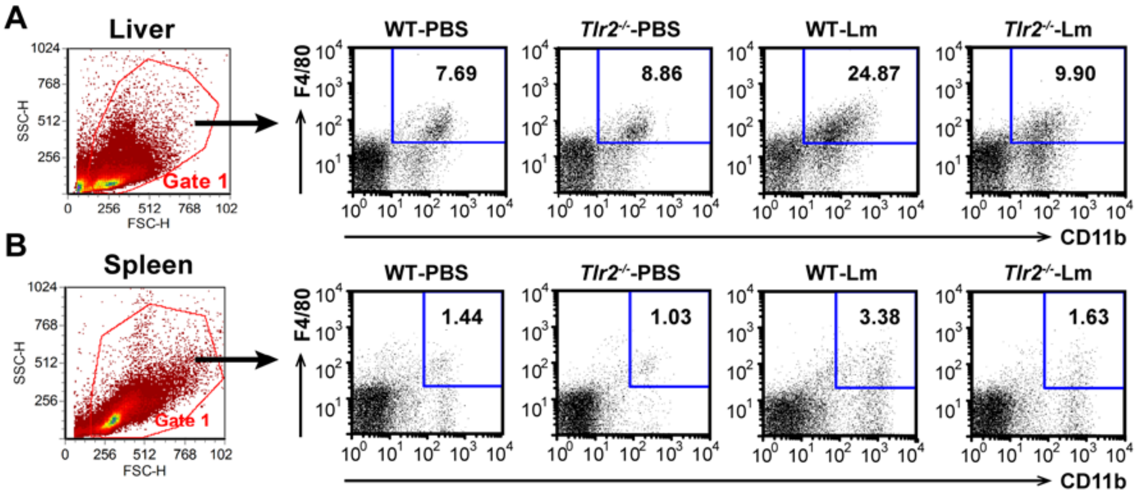

Supplementary Figure S2. The gating strategy of Mo/MΦ. (A-B) WT and *Tlr2*$^{-/-}$ mice were i.p. infected with 1×10^6 CFU of Lm. The gating strategy and the FACS blots of WT and *Tlr2*$^{-/-}$ mice in the liver (A) and spleen (B) 2 dpi (n = 5-6, one representative of two independent experiments).

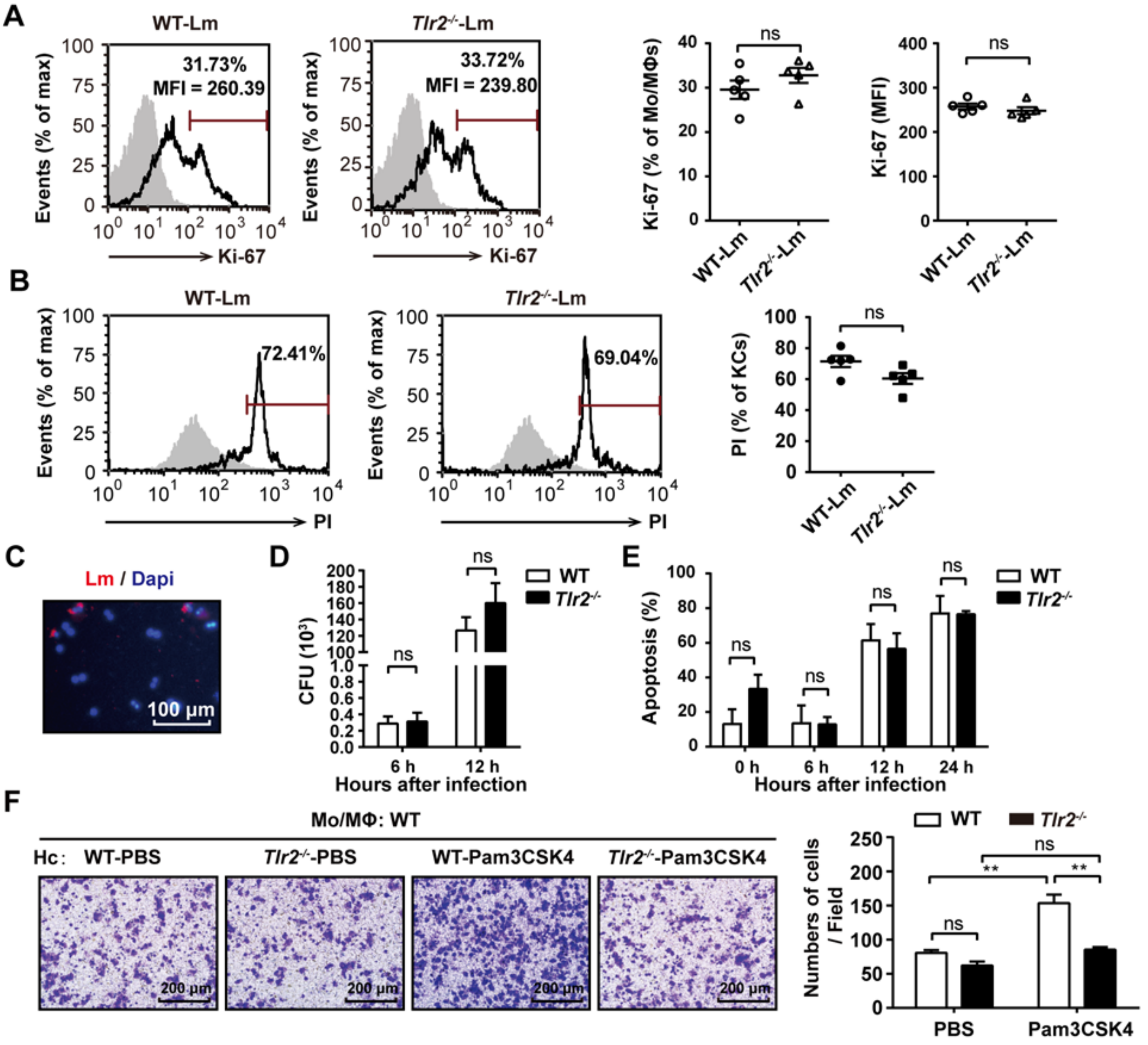

Supplementary Figure S3. TLR2 does not influence Mo/MΦ proliferation or hepatocyte apoptosis. (A) Age-matched WT and *Tlr2*$^{-/-}$ mice were i.p. infected with 1 × 10^6 CFU of Lm for two days. The level of Ki-67 expression on Mo/MΦs (CD11b$^+$ F4/80$^+$) in the liver was analyzed by FACS (n = 5). (B) WT and *Tlr2*$^{-/-}$ mice were i.p. infected with 1 × 10^6 CFU of Lm for 4 hours. The necroptosis level of Kupffer cells (F4/80$^+$ CD11b^{lo} Ly-6C^{lo}) in the liver was analyzed by FACS (n = 5). KCs, Kupffer cells. (C) Hepatocytes were isolated from WT mice and infected with Lm in vitro. Representative immunofluorescence image of Lm infected cells. Red, Listeria; blue, DAPI. One representative image of three independent experiments. (D-E) Hepatocytes were isolated from WT and *Tlr2*$^{-/-}$ mice and infected with Lm (MOI = 20:1) in vitro. The bacterial burden (D) was assessed at 6h and 12 h post-infection (three independent experiments). Hepatocyte apoptosis (both early and late apoptosis) (E) was detected by FACS at the indicated time points (three independent experiments). (F) Hepatocytes from WT or *Tlr2*$^{-/-}$ mice were seeded into the lower chamber and stimulated with 1 μg/mL Pam3CSK4 or PBS for 8 h, then the stimulation was removed and cultured in fresh DMEM for 12 h. WT Mo/MΦs were added to the inserts, and the photographs were taken after 4 h of migration (one representative image of three independent experiments). Data are presented as the mean ± SEM. **$p < 0.01$.

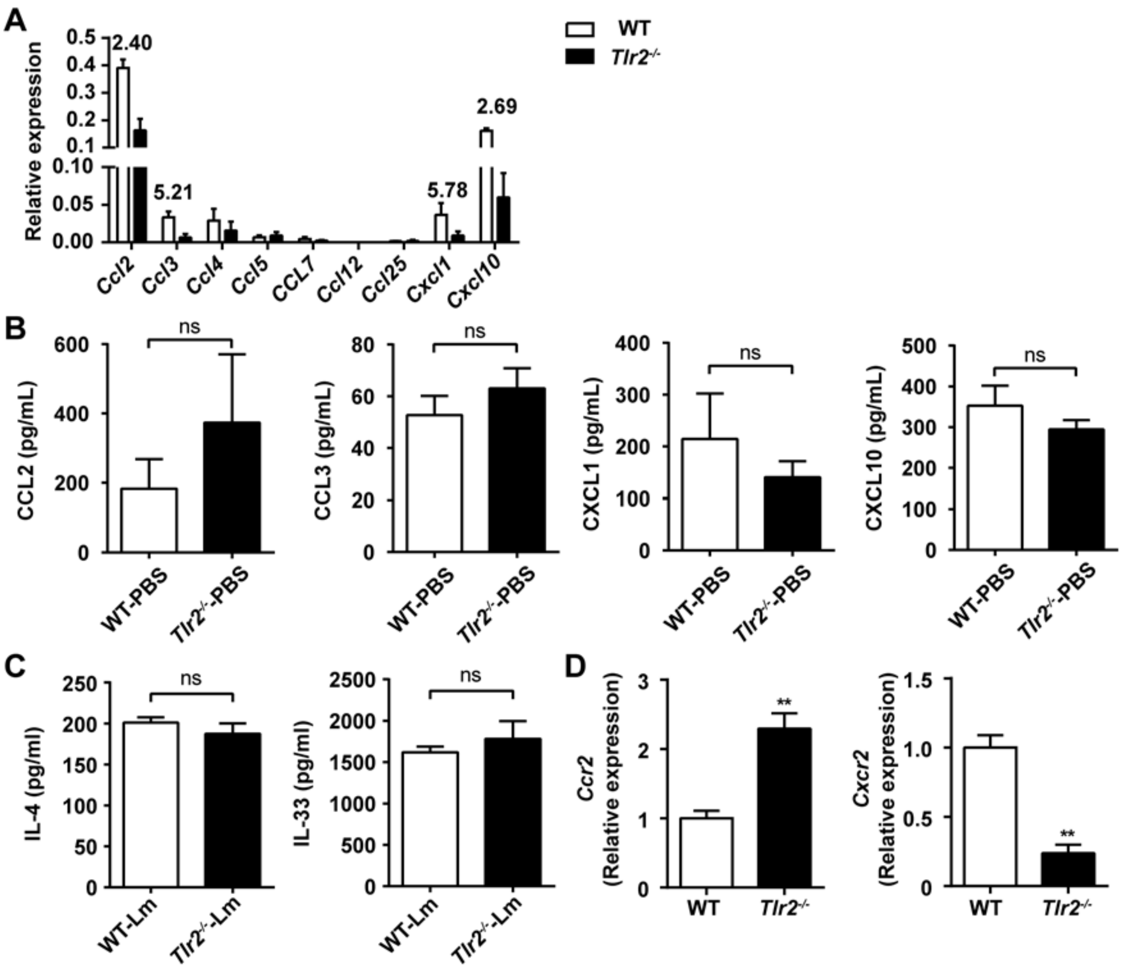

Supplemental Figure S4. TLR2 affects the expression of chemokine and chemokine receptors. (A) Hepatocytes were isolated from WT and *Tlr2*$^{-/-}$ mice and infected with Lm (MOI = 20:1) in vitro. Chemokine expression was measured by real-time PCR 12 h post infection (three independent experiments). (B) Hepatocytes were isolated from WT and *Tlr2*$^{-/-}$ mice and cultured for 12 h *in vitro*, respectively, and chemokine production was measured by ELISA (three independent experiments). (C) ELISA analysis of IL-4 and IL-33 in supernatants of the homogenized liver of Lm-infected WT and *Tlr2*$^{-/-}$ mice on 2 dpi (n = 5). (D) The expression of *Ccr2* and *Cxcr2* on Mo/MΦs obtained from WT and *Tlr2*$^{-/-}$ mice was measured by real-time PCR (n = 5). Data are presented as the mean ± SEM. **$p < 0.01$.

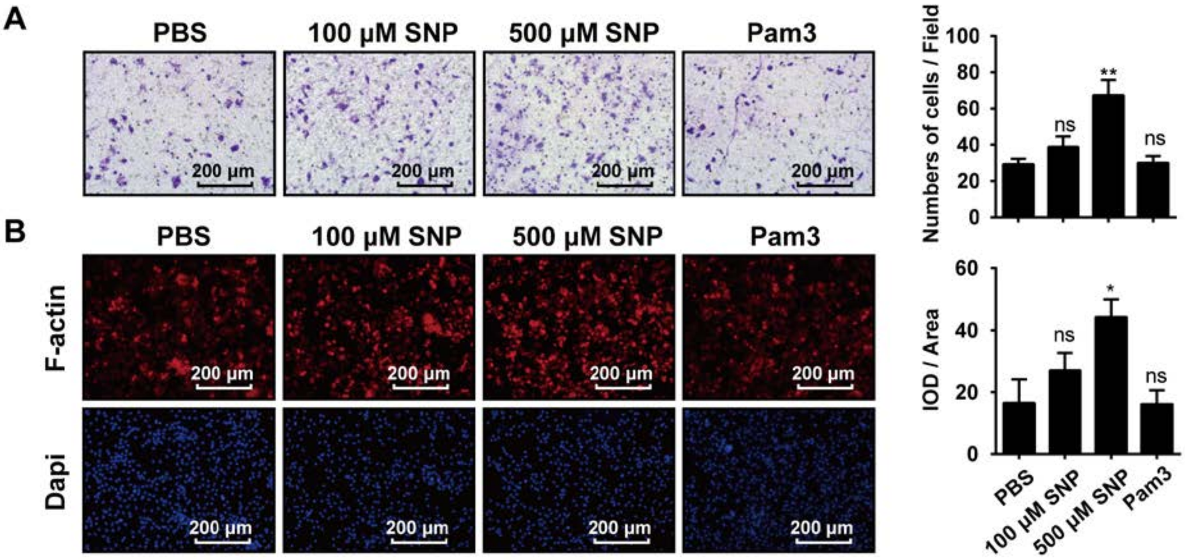

Supplementary Figure S5. NO pathway regulates F-actin polymerization in *Tlr2*$^{-/-}$ mice. (A) *Tlr2*$^{-/-}$ macrophages were added to the inserts and stimulated with SNP (NO donor), Pam3CSK4, or PBS as a control. Photographs were taken after 4 h of migration. (B) *Tlr2*$^{-/-}$ macrophages were settled on glass-bottom culture dishes and stimulated with SNP, Pam3CSK4, or PBS as a control for 1 h. F-actin was detected by rhodamine-phalloidin staining. Red, F-actin; blue, Dapi. The ratio of integrated optical density (IOD) to area of interest was calculated to assess the level of F-actin. Red, F-actin; blue, Dapi. Data are presented as the mean ± SEM of three independent experiments. *p < 0.05, **p < 0.01.

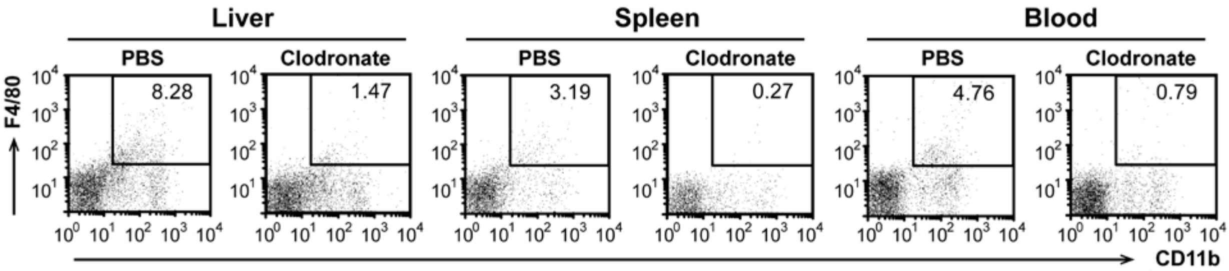

Supplementary Figure S6. The efficiency of macrophage depletion. The frequency of macrophages in the liver, spleen, and blood of WT mice intravenously injected with clodronate liposomes was detected by FACS on 2 days after injection (one representative of two independent experiments).

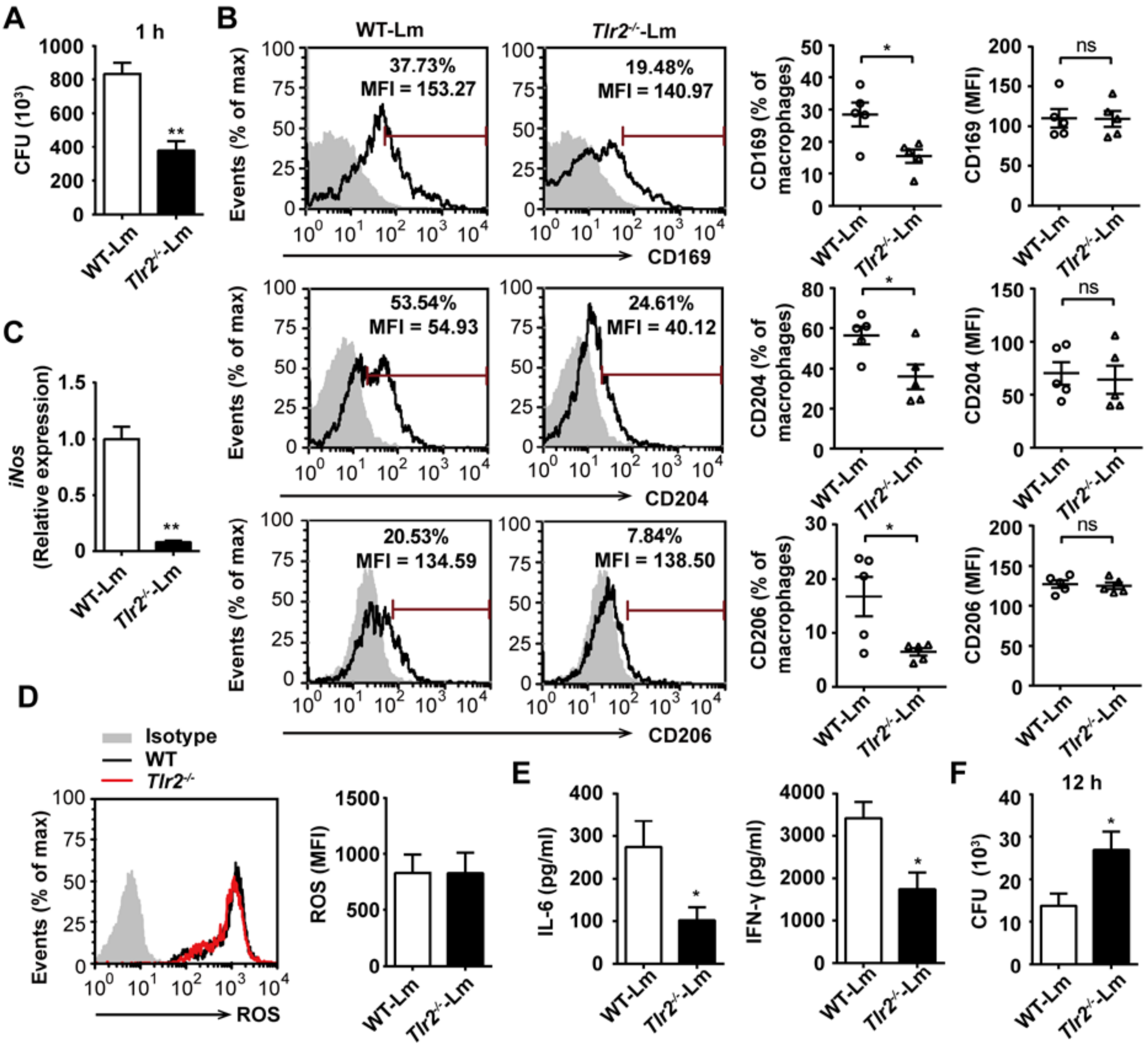

Supplemental Figure S7. TLR2 induces phagocytosis of Lm and promotes bacterial clearance in macrophages. (A) Peritoneal macrophages from WT and *Tlr2*$^{-/-}$ mice were isolated and infected with Lm (MOI = 10:1) in vitro. After 1 h incubation, the bacterial burden was assessed by determining CFU numbers. (B) WT and *Tlr2*$^{-/-}$ mice were i.p. infected with 1×10^6 CFU of Lm, and the expression of CD169, CD204, and CD206 on macrophages (CD11b$^+$ F4/80$^+$) in the liver was detected by FACS 2 dpi (n = 5, one representative of two independent experiments). (C-D) WT and *Tlr2*$^{-/-}$ mice were i.p. infected with 1×10^6 CFU of Lm, and peritoneal macrophages were isolated 1 dpi. The expression of *iNos* (C) was measured by real-time PCR, the level of ROS (D) was detected by FACS (n = 5). (E) The level of IL-6 and IFN-γ in the serum was measured by ELISA 2 dpi (n = 5). (F) Peritoneal macrophages were infected with Lm (MOI = 10:1) in vitro and incubated for 12 h. The bacterial burden was assessed by determining the CFU numbers. A and D-E, three independent experiments. Data are presented as the mean ± SEM. *$p < 0.05$, **$p < 0.01$.

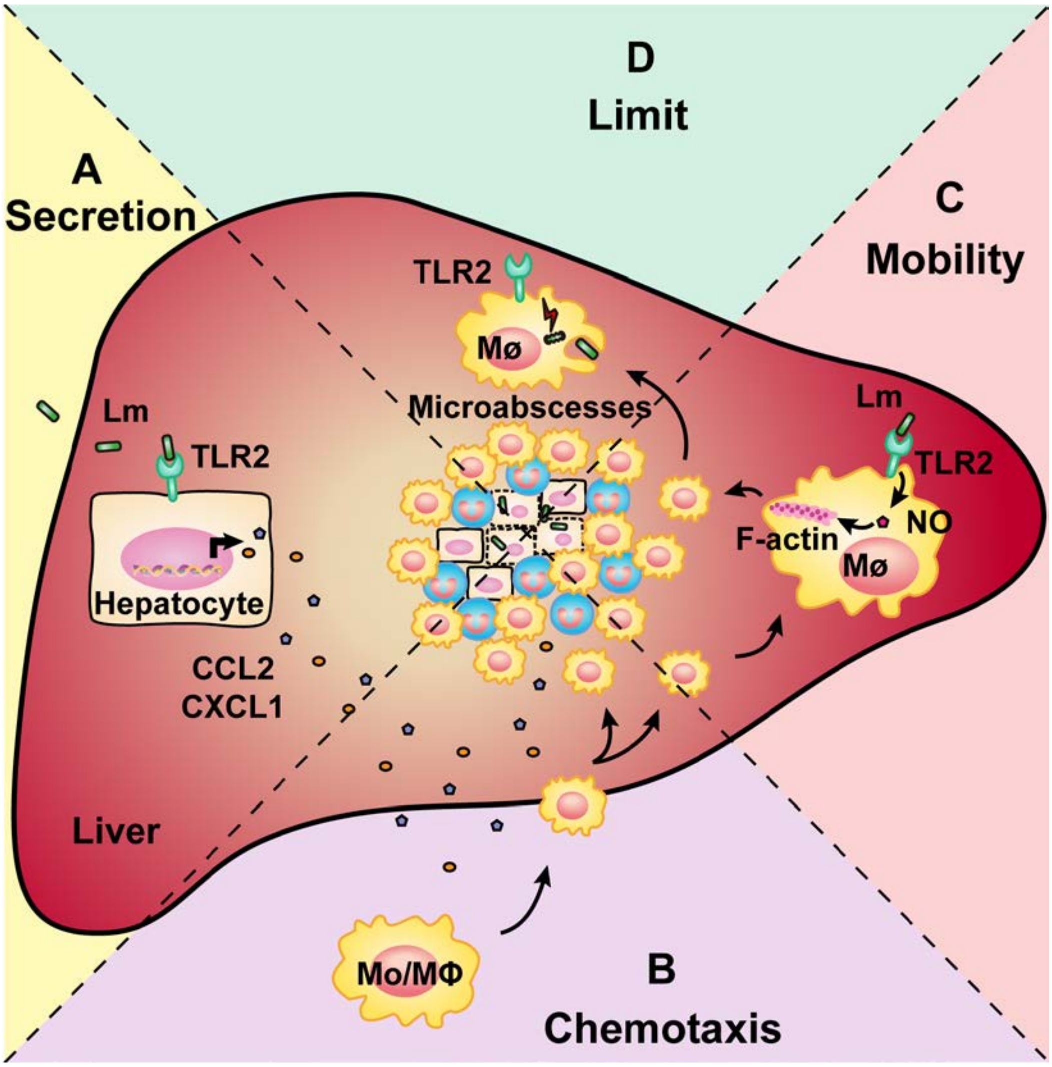

Supplemental Figure S8. Macrophages migrate into the liver by TLR2-induced chemotaxis and mobility to limit Lm spread. (A) TLR2 activation induces hepatocytes to secrete chemokines, including CCL2 and CXCL1 during Lm infection. (B) CCL2 and CXCL1 recruit monocytes and macrophages into the liver. (C) TLR2 promotes macrophage mobility through the TLR2/NO/F-actin pathway. (D) Macrophages participate in microabscess formation and limit Lm spread by TLR2-induced phagocytosis and bacterial clearance. Lm, *Listeria monocytogenes*; TLR2, Toll-like receptor 2; Neu, neutrophil; MΦ, macrophage; Mo/MΦ, monocyte/macrophage; F-actin, filamentous actin; NO, nitric oxide.

Supplementary Table S1. Sequence of primers used for qPCR in this study.

Gene	Forward Primer Sequence 5′→ 3′	Reverse Primer Sequence 5′→ 3′	Size (bp)
β-actin	AGAGGGAAATCGTGCGTGAC	CAATAGTGATGACCTGGCCGT	138
CCL2	ATTGGGATCATCTTGCTGGT	CCTGCTGTTCACAGTTGCC	108
CCL3	CTGCCCTTGCTGTTCTTCTC	CTTGGACCCAGGTCTCTTTG	231
CCL4	GCCCTCTCTCTCCTCTTGCT	GTCTGCCTCTTTTGGTCAGG	198
CCL5	GCTGCTTTGCCTACCTCTCC	TCGAGTGACAAACACGACTGC	104
CCL7	GCTGCTTTCAGCATCCAAGTG	CCAGGGACACCGACTACTG	135
CCL12	TGCCTCCTGCTCATAGCTACC	ACTGGCTGCTTGTGATTCTCC	151
CCL25	CCGGCATGCTAGGAATTATCA	GGCACTCCTCACGCTTGTACT	255
CXCL1	GCTTGAAGGTGTTGCCCTCAG	AGAAGCCAGCGTTCACCAGAC	201
CXCL10	GAAATCATCCCTGCGAGCCTATCC	GCAATTAGGACTAGCCATCCACTGGG	256
CCR2	GCTCTACATTCACTCCTTCCAC	ACCACTGTCTTTGAGGCTTG	133
CXCR2	TCTGCTCACAAACAGCGTCGTA	GAGTGGCATGGGACAGCATC	172
iNOS	GGCAAACCCAAGGTCTACGTT	TCGCTCAAGTTCAGCTTGGT	173
